# Telehealth in Radiation Oncology: Time to Refine, Not Just Expand

**DOI:** 10.7759/cureus.91860

**Published:** 2025-09-08

**Authors:** Join Y Luh

**Affiliations:** 1 Radiation Oncology, Providence St. Joseph Hospital, Eureka, USA

**Keywords:** radiation and medical oncology, teleassessment, telehealth appointments, telehealth policy, telemedicine and health outcomes

## Abstract

The COVID-19 pandemic accelerated the adoption of telehealth in radiation oncology, transforming on-treatment management visits (OTVs) into virtual encounters and expanding access, particularly in rural settings. A Medicare-based analysis by Patel et al. found that telehealth OTVs were associated with higher rates of ED visits, hospitalizations, and Medicare spending, raising concerns about unintended consequences of widespread telehealth use without adequate safeguards. While telehealth offers convenience and broader access, it may also compromise real-time clinical assessment and symptom management. These findings underscore the need for a risk-stratified, evidence-informed approach to telehealth policy in radiation oncology, one that prioritizes refinement and appropriate integration rather than unchecked expansion.

## Editorial

The COVID-19 pandemic catalyzed the widespread adoption of telehealth in the United States. Although telemedicine has existed since 1959, early systems were cumbersome, and face-to-face visits remained essential for delivering direct patient care. The 1990s brought renewed growth in telemedicine with the rise of the internet, but regulatory hurdles and limited insurance coverage constrained its use. Software platforms like Zoom and Microsoft Teams existed prior to the pandemic but were primarily used for business meetings and virtual conferences [1]. Once social distancing became the norm during the pandemic, Medicare and, eventually, private insurers, lifted restrictions on telehealth, including audio-only telephone visits (linked to CPT codes but previously unreimbursed). By the end of 2020, telemedicine had become standard for all evaluation and management services. This relaxation of restrictions also permitted weekly on-treatment management visits (OTVs) in radiation oncology (CPT 77427) to be conducted virtually.

As COVID-19 restrictions eased and in-person visits resumed, the convenience of telemedicine persisted. For patients, it reduced the burden of travel and parking and minimized time spent in crowded waiting rooms. Although patients still needed to travel to receive radiation, telemedicine allowed radiation oncologists to conduct OTVs without being physically onsite. A common scenario involved an oncologist at a main center remotely seeing patients at satellite clinics, particularly beneficial in rural areas with lower patient volumes. This arrangement reduced travel demands for both patients and physicians while helping to address chronic staffing shortages at satellite centers. On a broader scale, telemedicine has also been used for consults and follow-ups. During the pandemic, follow-up visits and consults were the most common uses of telemedicine among radiation oncologists (97% and 78%, respectively), compared with OTVs (36%) [2].

With ongoing debate about whether telemedicine for OTVs should remain permanent or be phased out, Patel et al. are to be commended for conducting the first outcomes-based analysis linking telehealth OTVs to downstream healthcare utilization, including ED visits and inpatient hospitalizations, between 2020 and 2022 [3]. Until now, much of the debate around telehealth lacked outcomes data. Assessing the safety of telehealth OTVs is difficult without understanding how widely they replace in-person physician assessments and what effect this substitution has on patient outcomes.

In this retrospective cohort study, Patel et al. examined nearly 370,000 Medicare radiation therapy OTV episodes and found that telehealth was used in 9.6% of 90-day episodes in 2020, declining to 5.5% in 2022. However, when telehealth was used, it accounted for about half of all weekly OTVs, indicating persistent adoption within certain practices [3].

The most striking finding was that patients managed with telehealth during radiation therapy had significantly higher rates of ED visits (38.2% vs. 30.2%) and hospitalizations (29.9% vs. 21.6%) compared with those managed entirely in person. After adjustment for clinical and demographic risk factors, telehealth remained a statistically significant independent predictor of ED use (OR 1.057) and inpatient stays (OR 1.080) [3]. These excess events translated into $16.3 million in additional Medicare spending over the two-year study period. If the observed excess costs associated with telehealth hold true across all radiation therapy episodes (369,570), not just the ~7% (~25,870) in which it was used, then universal telehealth adoption could result in nearly $230 million in additional Medicare spending in comparable time frames ($16,338,949 ÷ 25,870 ≈ $631 in excess cost per telehealth episode; $631 × 369,570 episodes ≈ $233,166,670).

Subset analysis by provider specialty revealed that among patients receiving concurrent chemotherapy, telehealth visits with medical oncologists were associated with excess ED and inpatient utilization. In contrast, telehealth visits with radiation oncologists did not increase acute care use in this cohort. However, in patients receiving radiation therapy alone, telehealth provided by radiation oncologists was linked to increased ED visits and hospitalizations [3]. Several explanations are plausible: in chemoradiation patients, medical oncologists may have been more concerned about abnormal lab values (which radiation oncologists are not directly responsible for), and radiation oncologists often defer systemic toxicity management to medical oncology in concurrent settings. This may have led to more acute care encounters when telehealth was used by medical oncologists. In patients receiving radiation alone, however, radiation oncologists conducting OTVs via telehealth may have defaulted to ED referrals for in-person assessment.

A personal anecdote illustrates the risks of relying solely on telecommunication. Years before COVID-19, I managed a breast cancer patient who had completed radiation therapy. Months later, she called, reporting shortness of breath. I ordered a chest X-ray, which was normal. When symptoms persisted, I prescribed azithromycin. Only when I asked her to come in did I notice her pallor, which led me to suspect anemia. A CBC confirmed this, and the anemia was later found to be due to bone marrow involvement from her breast cancer. Although this was a follow-up visit, a similar situation could have occurred during an OTV, where audiovisual technology would not have replicated the direct visual assessment that revealed the problem. I strongly support the telephone encounter (CPT 99441-3), but in this case, its limitations delayed the diagnosis.

What does this mean? It does not suggest that telehealth OTVs inherently cause waste or delays in care. However, the study highlights the unintended financial and clinical consequences of broad, unstructured adoption of telehealth in radiation oncology without safeguards. This analysis challenges the assumption that telehealth is an equivalent substitute for in-person care. While telehealth expands access, it may compromise surveillance, symptom management, or care coordination in our fragmented health system, with the most obvious limitation being the inability to perform a physical exam [2]. At a minimum, patients deserve a risk-stratified approach to telehealth policy.

Of course, this study has limitations. It is retrospective and based on Medicare claims, which do not capture patient satisfaction, nuanced clinical decision-making, or reasons for acute care use. Was the ED visit initiated by the patient or recommended by the physician? Did patients feel inadequately evaluated and seek in-person care themselves? Were physicians less confident in their telehealth assessments and therefore more likely to defer to the ED? These questions can only be addressed by prospective studies, which are needed to inform telehealth policy.

Nevertheless, this analysis contributes important data to an evolving discussion. Like rock and roll [4], telehealth is here to stay. But in radiation oncology, specifically for OTVs, it must be structured to address the clinical gaps it may unintentionally widen. Telehealth transformed cancer care delivery during the COVID-19 pandemic. In radiation oncology, however, it may also have introduced additional costs and risks. Moving forward, radiation oncologists must refine telehealth, not just expand it.

## References

[1] Luh JY Virtual telepresence. North Coast Physician.

[2] Damico NJ, Deshane A, Kharouta M (2022). Telemedicine use and satisfaction among radiation oncologists during the COVID-19 pandemic: evaluation of current trends and future opportunities. Adv Radiat Oncol.

[3] Patel KM, Thomas T, Heron DE, Bush A, Mantz CA (2025). Excess cost of telehealth use in radiation oncology: a medicare-based cohort study. Cureus.

[4] Danny & The Juniors "Rock 'n Roll is Here to Stay". https://www.youtube.com/watch?v=LNEj5FUHStE.

